# The emerging role of ¹⁸F-NaF PET/CT in osteoporosis: an emphasis on its application for the evaluation and management of lumbar spine osteoporosis

**DOI:** 10.1186/s13550-026-01411-1

**Published:** 2026-04-29

**Authors:** Jaskeerat Gujral, Om H. Gandhi, Amir A. Amanullah, Mert Marcel Dagli, Shashi B. Singh, Cyrus Ayubcha, Thomas J Werner, Mona-Elisabeth Revheim, William C. Welch, Abass Alavi

**Affiliations:** 1 Department of Radiology, Hospital of the University of Pennsylvania, 3400 Spruce St, Philadelphia, 19104 PA USA; 2 Department of Neurosurgery, Perelman School of Medicine, University of Pennsylvania, 801 Spruce Street, Philadelphia, 19107 PA USA; 3 Harvard Medical School, Boston, MA USA; 4 Division for Technology and Innovation, The Intervention Center, Oslo University Hospital, Rikshospitalet, Oslo, 0424 Norway; 5 Institute of Clinical Medicine, Faculty of Medicine, University of Oslo, Oslo, 0313 Norway

**Keywords:** Biomarkers, Lumbar spine, Osteoporosis, Positron emission tomography, Sodium fluoride

## Abstract

**Background:**

Osteoporosis is a chronic skeletal disorder characterized by reduced bone mineral density and disrupted bone microarchitecture, affecting over 200 million individuals worldwide. The lumbar spine, containing the largest volume of metabolically active trabecular bone, is particularly vulnerable to osteoporotic degeneration and compression fractures. This narrative review examines recent advances in imaging modalities for lumbar spine osteoporosis assessment, emphasizing the diagnostic utility and emerging clinical applications of ¹⁸F-sodium fluoride (NaF) positron emission tomography/computed tomography (PET/CT). A comprehensive narrative review was conducted, synthesizing findings from pivotal studies investigating conventional imaging methods and newer PET-based technologies for osteoporosis evaluation. Particular focus was given to studies utilizing quantitative and kinetic PET biomarkers for assessing bone metabolic activity with ¹⁸F-NaF.

**Main body:**

While dual-energy X-ray absorptiometry (DXA) remains the clinical standard for bone mineral density assessment, it has significant limitations including poor spatial resolution, lack of three-dimensional capability, and inability to differentiate cortical from trabecular bone. In contrast, ¹⁸F-NaF PET/CT demonstrates superior image quality, rapid tracer kinetics, and quantitative assessment of regional osteoblastic activity. Studies show strong correlations between ¹⁸F-NaF uptake and bone turnover markers, mineral density measurements, and therapeutic response. Kinetic modeling approaches provide detailed insights into bone remodeling dynamics, supporting personalized treatment planning and prognostic assessment. Diagnostic performance studies report area under the receiver operating characteristic curves as high as 0.96 for osteoporosis detection when evaluated against DXA-derived BMD, though no study has yet compared both modalities against an independent gold standard such as fracture outcomes.

**Conclusion:**

¹⁸F-NaF PET/CT offers optimal clinical applications for early treatment response monitoring, evaluation of patients with discordant clinical risk and DXA findings, pre-surgical assessment in patients with borderline bone density, and investigation of complex metabolic bone disorders. Ideally, ¹⁸F-NaF PET/CT should be utilized in a complementary fashion to DXA. Primary barriers to clinical adoption include cost, limited accessibility, and absence of standardized kinetic modeling protocols. Future research should focus on establishing reference ranges across age and sex demographics, validating fracture prediction models, and determining cost-effectiveness thresholds for specific clinical scenarios such as high-risk patients with discordant DXA and fracture history.

## Background

### Introduction

#### Introduction to osteoporosis and lumbar osteoporosis

Osteoporosis is one of the most significant skeletal disorders of the modern era, affecting over 200 million individuals worldwide and characterized by reduced bone mineral density and disrupted bone microarchitecture that substantially increases fracture risk [1]. Osteoporosis follows a pronounced age-related pattern, with its prevalence exceeding 70% among individuals over 80 years of age. As populations continue aging globally, epidemiological projections indicate a three-fold increase in osteoporotic fractures [2, 3]. Additionally, the clinical and economic burden is staggering, with approximately two million osteoporotic fractures occurring annually in the United States alone, resulting in an estimated $17 billion in healthcare expenditure [4, 5]. Demographic patterns reveal that while Black populations exhibit lower incidence rates compared to White populations, post-diagnosis fracture risks remain comparable across racial groups. Notably, projections indicate that over half of postmenopausal White women will sustain an osteoporotic fracture during their lifetime [6]. Collectively, these population statistics underscore the increasing clinical burden and public health relevance of osteoporosis, warranting improved diagnostic strategies to facilitate earlier disease detection, prevent rapid disease progression, and better optimize treatment monitoring.

Osteoporosis occurs in two primary forms: primary osteoporosis, which results from hormonal changes during natural aging (often seen in women with estrogen deficiency after they reach menopause), and secondary osteoporosis, which arises from underlying medical conditions or medication side effects, with glucocorticoids being the most common medication that leads to osteoporosis and increased fracture risk [7, 8]. Both types involve an imbalance in bone remodeling that decreases bone mass and increases fracture risk.

The lumbar spine is particularly susceptible to osteoporotic damage due to its high metabolic activity and continuous mechanical load from supporting the weight of the upper body. This persistent mechanical stress, coupled with age-related bone loss, contributes to the lumbar spine’s involvement in vertebral compression fractures [9]. Furthermore, the spine contains the largest volume of metabolically active trabecular bone in the body [10]. Consequently, the loss of these trabeculations is a hallmark feature of osteoporosis [11]. Age-related bone deterioration weakens vertebral integrity, and this increases the risk of fractures and spinal deformities even under normal physiological loading conditions [12]. Postmenopausal women are at a significantly increased risk of spinal osteoporosis due to accelerated axial bone loss following menopause [13]. Moreover, the evaluation of vertebral bone density can be confounded due to age-related changes such as aortic calcification, which creates artifactual density increases on imaging studies, or hypertrophic spinal changes including osteophytes and facet joint sclerosis, which can mask true bone mineral density measurements by adding extraneous calcified tissue that is not representative of vertebral body trabecular bone health. These confounding factors were particularly problematic in earlier DXA studies using planar imaging techniques, though modern volumetric imaging methods like quantitative computed tomography (QCT) can better differentiate trabecular bone from cortical and degenerative changes [14–18].

Bisphosphonates remain the cornerstone of pharmacologic management of osteoporotic fractures as they are potent inhibitors of osteoclast mediated bone resorption. These agents reduce bone turnover, enhance bone mineral density, and lower the risk of both vertebral and non-vertebral fractures [19–23]. Alendronate, a widely prescribed bisphosphonate, exerts its effects by binding to hydroxyapatite in bone and promoting osteoclast apoptosis, thereby suppressing bone resorption. However, assessing treatment efficacy often requires advanced imaging capabilities which extend beyond traditional methods.

#### Diagnostic modalities

Several imaging modalities are currently used in the clinical diagnostic work-up for osteoporosis, including dual-energy X-ray absorptiometry (DXA) and nuclear medicine radiotracers, such as technetium-99 m methylene diphosphonate (⁹⁹ᵐTc-MDP) and fluoride-18 sodium fluoride (¹⁸F-NaF). Despite their widespread use, both DXA and ⁹⁹ᵐTc-MDP imaging have notable limitations that impair diagnostic accuracy. DXA, in particular, is hindered by technical constraints such as limited spatial resolution to evaluate bone microarchitecture, absence of three-dimensional capability, and inability to distinguish between cortical and trabecular bone [9, 24]. Moreover, DXA’s diagnostic reliability is frequently compromised by errors in scan acquisition, data processing, analysis, and result interpretation, which can further hinder clinical decision-making [25, 26]. The World Health Organization has established a standardized classification system for bone mineral density based on T-scores derived from DXA measurements. This classification defines three distinct categories: normal bone density (T-score ≥ -1.0), osteopenia (T-score between − 1.0 and − 2.5), and osteoporosis (T-score ≤ -2.5). While this classification system provides a framework for clinical diagnosis and risk stratification, it underscores the limitations of relying solely on DXA-derived bone mineral density measurements for comprehensive osteoporosis assessment [27]. Furthermore, several studies have questioned the accuracy of DXA-derived BMD values [28–30].

Aside from DXA, ⁹⁹ᵐTc-MDP bone scintigraphy with single-photon emission computed tomography/computed tomography (SPECT/CT) carries several limitations. As a bisphosphonate analog, ⁹⁹ᵐTc-MDP undergoes slow metabolic clearance and is highly dependent on renal excretion [31, 32]. In patients with age-related renal impairment, relying on renal clearance may result in overestimation of osteoblastic activity due to delayed excretion and increased retention of the ⁹⁹ᵐTc-MDP radiotracer. Additionally, ⁹⁹ᵐTc-MDP’s affinity for plasma proteins can interfere with quantitative accuracy [33]. ⁹⁹ᵐTc has a relatively long half-life of approximately 6 h, which is longer than the half-life of the most used positron emitter ¹⁸F-NaF (110 min) [34]. Radiation doses vary among these imaging modalities. The radiation doses for plain films have been noted to be approximately 0.26 millisievert (mSv) in the lumbar spine, while DXA has been reported to range from 0.02 to 0.06 mSv for the lumbar spine [35, 36]. For ⁹⁹ᵐTc-MDP bone scintigraphy, the effective whole body radiation dose is approximately 4 to 6 mSv/25 mCi, and SPECT/CT adds additional radiation dosages depending on institutional protocols. Furthermore, ¹⁸F-NaF PET/CT may expose patients to 7 to 10 mSv [37]. In summary, DXA has the least radiation exposure, followed by plain film X-rays, then ⁹⁹ᵐTc-MDP bone scintigraphy with ¹⁸F-NaF PET/CT carrying the greatest radiation exposure. For instance, a standard 370-MBq ¹⁸F-NaF dose results in approximately 8.9 mSv to an adult patient, representing 50–70% higher exposure than typical ⁹⁹ᵐTc-MDP doses. However, modern PET scanners allow materially lower activities (as low as 90 MBq), making the dose of radiation exposure comparable to, or even lower than, ⁹⁹ᵐTc-MDP.

Although DXA remains the clinical gold standard for diagnosing osteoporosis, DXA is limited in its ability to fully characterize bone quality and assess treatment response. These limitations have driven clinical interest in advanced imaging modalities, particularly positron emission tomography (PET) integrated with CT, which offer more comprehensive molecular, functional and structural evaluation of osteoporosis. Among novel imaging tools, ^18^F-NaF PET offers unique molecular insight into bone metabolism, aiding in the detection and characterization of various osseous pathologies [24].

#### Applications of ¹⁸F-NaF PET in osteoporosis

¹⁸F-NaF was initially introduced in the 1960s but was subsequently supplanted by ⁹⁹ᵐTc-MDP due to the latter’s superior compatibility with gamma cameras [24, 38, 39]. Although ¹⁸F-NaF received U.S. Food and Drug Administration (FDA) approval for bone scintigraphy in 1972, it was withdrawn in 1975 for non-clinical reasons. The emergence of whole-body PET scanners in the 1990s facilitated the reintroduction of ¹⁸F-NaF into clinical practice, allowing for high-resolution, high-contrast bone imaging [40]. More recently, the widespread availability of PET imaging systems, including both PET/CT and PET/MRI platforms, has further enhanced clinical adoption of ¹⁸F-NaF imaging.

¹⁸F-fluoride is synthesized by proton irradiation of ¹⁸O-water using cyclotrons with tantalum targets [41, 42]. The irradiated solution is purified through sequential cation (H+ form) and anion (HCO_3_− form) exchange cartridges to isolate the ¹⁸F-fluoride. The ¹⁸F-fluoride is then eluted with sterile normal saline and passed through a filter into a sterile multidose vial, where the quality tests are done [42].

The physical half-life of ¹⁸F-NaF is 109.7 min and decays into stable ^18^O with ejection of a positron from the nucleus. This ejected positron annihilates an electron, producing two 511-keV photons [40].

^18^F-NaF offers several key advantages for imaging bone disorders, particularly osteoporosis. This radiotracer provides unique molecular-level perspectives on underlying metabolic alterations that contribute to bone diseases such as osteoporosis. A major advantage of ^18^F-NaF is its minimal protein binding, which facilitates high first-pass extraction and rapid clearance from soft tissues [43]. These pharmacokinetic properties allow for PET image acquisition as early as 30–45 min, although for full quantification, 60 min is recommended [44]. With complete clearance of NaF from the circulation by 45 min, PET images can be obtained with reduced background activity, ultimately enhancing image quality [45–47]. After injection, ¹⁸F-NaF diffuses through capillaries into bone extracellular fluid, where fluoride ions exchange with hydroxyl groups on hydroxyapatite minerals at the bone surface [24, 48]. ¹⁸F-NaF’s high affinity for regions undergoing active bone remodeling enables detection of osteoblastic activity and regional blood flow, making it a sensitive biomarker for metabolic bone disease [40]. The degree of ¹⁸F-NaF radiotracer uptake corresponds to the availability of binding sites located on bone, which allows for quantitative assessment of biochemical processes contributing to bone pathology [24]. The tracer freely diffuses across membranes and undergoes rapid plasma clearance and renal excretion, with less than 10% of the injected dose remaining in blood circulation after 45 min post injection [40, 45–47].

In osteoporosis and other similarly related bone disorders, ¹⁸F-NaF serves as a sensitive marker of bone turnover by directly reflecting osteoblastic activity [49]. Osteoblastic activity can be directly measured using ¹⁸F-NaF because radioactive fluoride ions emitted during positron decay are incorporated into hydroxyapatite at sites of active bone formation, generating detectable photons through positron-electron annihilation [44]. ¹⁸F-NaF PET provides molecular-level insight into osteoblast dysfunction and bone remodeling, making it a valuable imaging tool for detecting and monitoring osteoporotic changes in metabolic bone disease.

Beyond ¹⁸F-NaF, several other bone-seeking radiopharmaceuticals have been investigated for evaluating skeletal metabolism in the context of osteoporosis. Calcium-45 (Ca-45) and calcium-47 (Ca-47) are radioactive calcium isotopes that directly trace calcium incorporation into the bone matrix, providing a measure of bone formation; however, their limited availability, unfavorable dosimetry, and long half-lives have restricted their clinical application [50, 51]. Strontium-85 (Sr-85), a calcium analog that is incorporated into hydroxyapatite at sites of active bone formation, has been used to assess regional bone turnover but is limited by suboptimal imaging characteristics and prolonged skeletal retention [52, 53]. Phosphorus-32 (P-32) accumulates in metabolically active bone but primarily has therapeutic applications in polycythemia vera and bone metastases rather than diagnostic imaging of osteoporosis [54, 55]. ¹⁸F-fluorodeoxyglucose (¹⁸F-FDG), the most widely used PET tracer in oncology, reflects glucose metabolism and can detect inflammatory and cellular activity within the bone marrow microenvironment [56]. While ¹⁸F-FDG has demonstrated utility in detecting metabolically active bone marrow changes associated with osteoporosis, it fundamentally differs from ¹⁸F-NaF in its mechanism of uptake: ¹⁸F-FDG reflects cellular glucose metabolism, particularly of marrow cells and inflammatory infiltrates, whereas ¹⁸F-NaF is incorporated directly into the bone mineral matrix and specifically reflects osteoblastic activity and regional bone blood flow [40, 57]. Thus, ¹⁸F-NaF provides a more direct and specific assessment of bone remodeling dynamics compared to ¹⁸F-FDG, making it the preferred PET radiotracer for evaluating bone formation in osteoporosis.

This review synthesizes current evidence regarding imaging modalities for osteoporosis, with particular emphasis on the utility of ¹⁸F-NaF PET/CT in evaluating osteoporotic changes within the lumbar spine. We critically examine the diagnostic performance, quantitative capabilities, and therapeutic monitoring applications of ¹⁸F-NaF PET/CT compared to conventional imaging approaches. The adoption of advanced quantitative imaging technologies in clinical workflows will enable more precise diagnosis and management of patients with osteoporosis.

## Main text

### Quantitative assessment using ^18^F-NaF-PET

Established protocols for ^18^F-NaF PET enable clinicians to obtain quantitative parameters that provide insight into bone metabolism and remodeling processes. These measurements range from simple semi-quantitative metrics to sophisticated kinetic modeling approaches. The most commonly used metric is the standardized uptake value (SUV), which represents the uptake of radiotracer in a specific region normalized to the injected dose and body weight [58]. SUV_mean_ and SUV_max_ provide simple, semi-quantitative evaluation of tracer uptake with relatively low precision error. However, it is critical to recognize that SUV measurements represent composite parameters influenced by several distinct physiological factors beyond osteoblastic activity alone. Specifically, ^18^F-NaF uptake depends on both regional blood flow (tracer delivery) and the available surface area of hydroxyapatite for binding. Consequently, SUV values reflect bone perfusion, bone volume, trabecular microarchitecture, and metabolic activity simultaneously rather than representing a pure measure of bone formation [59, 60].

This composite nature of SUV measurements explains apparently paradoxical findings in the literature. In cross-sectional observational studies, lower SUV values correlate with lower bone mineral density, as osteoporotic bone exhibits reduced trabecular volume and surface area available for fluoride binding, along with decreased osteoblastic activity [61]. However, in interventional studies evaluating bisphosphonate therapy, SUV values may decrease despite stable or increasing BMD [62]. This occurs because bisphosphonates primarily suppress osteoblastic activity and bone turnover rather than immediately affecting bone volume or blood flow. The reduction in SUV following bisphosphonate treatment reflects the intended pharmacologic suppression of bone remodeling, not deterioration of bone quality. Conversely, anabolic agents like teriparatide increase SUV by stimulating osteoblastic activity even before substantial changes in bone volume occur.

These observations reveal a fundamental limitation of static SUV measurements: they cannot independently quantify the relative contributions of blood flow, bone surface area, and metabolic activity to the observed signal. This limitation has important implications for longitudinal monitoring, as changes in SUV may reflect alterations in any or all these components. Furthermore, technical factors including image reconstruction methods and the timing of image acquisition can influence SUV values, warranting caution when applying these parameters in research studies involving comparisons between patient cohorts or in longitudinal assessments [59, 60]. The question of whether bone volume-adjusted SUV measures should be implemented remains an area of active investigation, though no standardized normalization approach has been established in clinical practice.

To address the inherent limitations of SUV measurements and provide more direct insights into bone physiology, kinetic modeling approaches have been developed to separately quantify the rates of specific physiological processes. These models mathematically separate the composite PET signal into distinct rate constants representing tracer delivery from plasma to bone (K₁), efflux back to plasma (k₂), and binding within bone tissue (k₃) [45, 63–65]. The net influx rate constant (Ki) derived from these models represents the plasma clearance of fluoride to bone mineral and provides a more specific measure of bone formation that is less confounded by variations in blood flow or bone volume than static SUV measurements.

Two kinetic modeling frameworks have been applied in research settings and select clinical centers. The Hawkins model is a three-compartment model that employs dynamic scan methodology utilizing time-activity curves and arterial input functions to determine all individual rate constants (K₁, k₂, k₃, and k₄). This approach provides the most complete physiological information by separately quantifying delivery, binding, and release kinetics, but requires dynamic imaging protocols and arterial blood sampling. In contrast, Patlak’s graphical model is a computational approach that determines Ki through graphical analysis of the data, assuming irreversible trapping of the tracer during the imaging period [66, 67]. The Patlak method offers a more practical approach for routine clinical implementation, as it provides reliable kinetic parameter estimation without requiring arterial blood sampling or multi-compartmental fitting procedures.

Accurate determination of the arterial input function is a critical requirement for kinetic modeling and can be obtained through several approaches with varying levels of invasiveness and practicality. While arterial blood sampling remains the gold standard, it is invasive and impractical for clinical use, though it continues to serve an important role in research studies and method validation [45, 68]. Image-derived input functions offer a non-invasive alternative by extracting time-activity curves from large blood vessels, although this approach requires corrections for partial volume effects and spillover from surrounding tissues [69, 70]. Population-based input functions provide a practical compromise for clinical applications by using averaged curves from previous arterial sampling studies, which are then scaled to individual patients using readily obtainable parameters such as late venous blood samples [71]. For ¹⁸F-NaF specifically, Blake et al. developed a semi-population method that combines standardized early-phase curves with individual venous samples, enabling Ki estimation at multiple skeletal sites using short static scans without requiring dynamic imaging [72].

The clinical value of kinetic modeling over simple SUV measurements is particularly evident in treatment monitoring. As demonstrated by Frost et al., risedronate therapy resulted in an 18.4% reduction in vertebral Ki that paralleled the decline in bone-specific alkaline phosphatase, directly reflecting suppression of bone formation [73]. Simultaneously, the study observed a significant increase in k₂ (the rate constant for fluoride efflux from bone), indicating altered tracer kinetics beyond simple uptake reduction. These distinct changes in individual rate constants would not be captured by SUV measurements alone, which aggregate all these processes into a single composite value. Similarly, the precision study by Al-Beyatti et al. demonstrated that Ki measurements, particularly those derived using the Patlak method, provide reproducible quantitative assessment suitable for longitudinal monitoring, with coefficient of variation values ranging from 9.2% for SUV to 11.7% for Patlak-derived Ki [74].

While traditional dynamic imaging restricts assessment to single bed positions due to limited scanner field of view, multi-timepoint protocols have been validated as an alternative approach. These protocols use brief static acquisitions at different sites (typically 5–10 min at 30, 45, and 60 min post-injection), allowing assessment of clinically relevant locations including the spine and hips with a single tracer injection [75]. Looking forward, emerging total-body PET scanners with extended axial coverage may enable simultaneous dynamic assessment of multiple skeletal sites while substantially reducing radiation doses [76].

In summary, while SUV measurements provide accessible semi-quantitative assessment of bone metabolism, their interpretation requires careful consideration of the underlying physiological and technical factors that influence tracer uptake. Kinetic modeling approaches, though more technically demanding, may offer superior mechanistic insight by separately quantifying the components of bone metabolism, making them particularly valuable for treatment monitoring and research applications where understanding the biological basis of observed changes is essential. The apparent discrepancy between observational correlations (lower SUV with lower BMD) and interventional effects (lower SUV with effective bisphosphonate therapy) reflect the different physiological states being measured: chronic bone loss versus acute therapeutic suppression of bone turnover.

### Diagnostic applications of ¹⁸F-NaF PET/CT

Several studies have investigated the role of ¹⁸F-NaF PET/CT in assessing osteoporosis of the lumbar spine (Table 1). In a comprehensive retrospective study, Huang et al. analyzed 796 lumbar vertebrae from 199 patients with a history of cancer who underwent both ¹⁸F-NaF PET/CT and lumbar DXA scanning [61]. Patients were categorized into three groups based on DXA derived BMD: normal BMD, osteopenic, and osteoporotic. The study found significantly reduced ¹⁸F-NaF uptake in osteoporotic L1-L4 vertebrae compared to those with osteopenia and normal BMD. On a vertebra-level analysis, maximum standardized uptake values (SUVₘₐₓ) were 8.13 ± 1.28 for normal BMD vertebrae, compared to 6.61 ± 1.01 for osteopenic (*P* < 0.0001) and 4.82 ± 1.01 for osteoporotic vertebrae (*P* < 0.0001). A strong positive correlation was observed between ¹⁸F-NaF uptake and BMD across all lumbar vertebrae. The diagnostic performance of ¹⁸F-NaF PET/CT was robust, with an area under the receiver operating characteristic (AUROC) curve of 0.96 for identifying osteoporosis and 0.83 for osteopenia. This study highlights the high diagnostic performance when ¹⁸F-NaF PET/CT was evaluated against DXA derived BMD. However, the study did not conduct a face-to-face comparison through the utilization of an independent gold standard.

**Table 1:** Comparison of nuclear medicine modalities for osteoporosis assessment

Modality	Physical half-life	Radiation dose (lumbar spine)	Imaging time post-injection	Spatial resolution	Practical applications	Advantages	Limitations	Analytical techniques
DXA	N/A	0.02-0.06 mSv	Immediate	Limited (2D planar)	Bone mineral density measurement; Osteoporosis diagnosis; Fracture risk assessment	Lowest radiation exposure; Widely available; Low cost; Established clinical standard	Poor spatial resolution; No 3D capability; Cannot differentiate cortical from trabecular bone; Susceptible to artifacts from calcifications	T-score calculation; BMD measurement (g/cm²)
⁹⁹ᵐTc-MDP SPECT/CT	6 hours	4-6 mSv (bone scan) + CT component	2-4 hours	~8-12 mm	Fracture detection; Metastasis evaluation; Bone turnover assessment	Established clinical use; Whole-body imaging capability; CT co-registration available	Long half-life requiring delayed imaging; Plasma protein binding affects quantification; Lower spatial resolution than PET; Dependent on renal clearance	Visual interpretation; Semi-quantitative uptake ratios; SPECT quantification
¹⁸F-NaF PET/CT	110 minutes	7-10 mSv (standard dose); ~3-5 mSv (modern scanners)	30-60 minutes	~4-5 mm	Regional bone metabolism quantification; Osteoporosis detection; Treatment response monitoring; Metastasis detection	Superior spatial resolution; Rapid tracer kinetics; Minimal protein binding; High first-pass extraction; Quantitative assessment of osteoblastic activity; Molecular-level insights	Higher radiation exposure (standard protocols); Limited availability; Higher cost; Requires PET scanner; Renal impairment affects pharmacokinetics	SUV (SUVₘₐₓ, SUVₘₑₐₙ); Kinetic modeling (Hawkins model); Patlak graphical analysis; Ki determination; Compartmental analysis (K₁, k₂, k₃, k₄)
QCT	N/A	1.5-3 mSv	Immediate	High (3D volumetric)	Volumetric BMD measurement; Differentiation of cortical and trabecular bone; Finite element analysis	3D assessment; Separates trabecular from cortical bone; No radiotracer required	Higher radiation than DXA; More expensive than DXA; Less standardized protocols	Volumetric BMD (mg/cm³); Hounsfield unit analysis; Finite element modeling

Comprehensive comparison of imaging modalities used for osteoporosis assessment in the lumbar spine. DXA = dual-energy X-ray absorptiometry; SPECT/CT = single-photon emission computed tomography/computed tomography; ⁹⁹ᵐTc-MDP = technetium-99m methylene diphosphonate; PET/CT = positron emission tomography/computed tomography; ¹⁸F-NaF = fluoride-18 sodium fluoride; QCT = quantitative computed tomography; BMD = bone mineral density; SUV = standardized uptake value; Ki = net influx rate constant; K₁, k₂, k₃, k₄ = compartmental rate constants

The role of ¹⁸F-NaF PET/CT parameters, specifically SUV and K_i_ in assessing bone metabolic activity was evaluated utilizing 63 ¹⁸F-NaF PET/CT scans of the L1-L4 vertebrae, alongside Hounsfield units (HUs) from the corresponding CT images [77]. Areal BMD data were obtained from DXA scans, and bone mineral apparent density (BMAD) was calculated to estimate volumetric BMD. The results revealed that HUs demonstrated the strongest correlation with both SUV (*P* < 0.0001) and K_i_ (*P* < 0.0005), while BMAD exhibited the weakest association. Jassel et al.’s findings support the utility of ^18^F-NaF PET/CT as a reliable modality for quantifying bone metabolic activity in the lumbar spine.

Another study has evaluated osteoporosis in a clinically distinct cohort of multiple myeloma patients using ¹⁸F-NaF PET/CT data from the FULIMA trial [78]. Methodology involved examining L1-L4 vertebral trabecular bodies in 33 patients, and their analysis revealed a significantly positive correlation between HU values and SUVₘₑₐₙ (*p* < 0.0001) and negative correlations between age and both HUs (*r* = -0.59, *p* < 0.0003) and SUVₘₑₐₙ (*p* = 0.002). Among multiple myeloma patients, those with osteoporosis demonstrated approximately a 29.1% reduction in ¹⁸F-NaF uptake (SUVₘₑₐₙ = 4.96 ± 1.46) compared to multiple myeloma patients without osteoporosis (SUVₘₑₐₙ = 7.0 ± 1.48). The predictive value of ¹⁸F-NaF PET/CT for identifying reduced osteoblastic activity was supported by an AUC of 0.857 (*p* = 0.002), indicating high accuracy for lumbar spine osteoporosis.

**Fig. 1:**
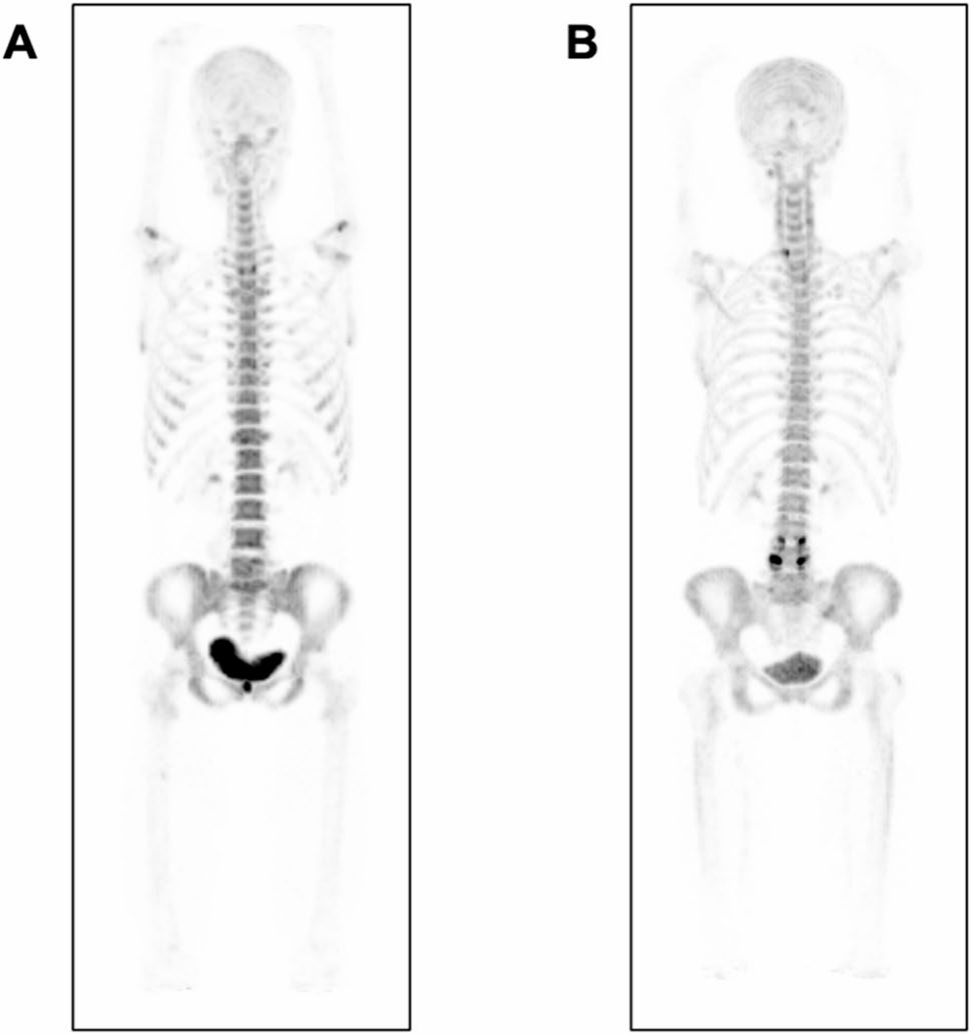
Age-related differences in ^18^F-NaF-PET uptake in the skeleton. Maximum intensity projection (MIP) ^18^F-NaF-PET images after injection from two healthy female subjects: (**A**) a 26-year-old and (**B**) a 62-year-old. The older subject demonstrates qualitatively reduced tracer uptake in metabolically active regions including the spine, pelvis, and proximal femora, consistent with age-associated decline in osteoblastic activity and bone turnover. These visual differences reflect biological variation in skeletal metabolism rather than differences in image windowing. While the original published figure does not include an SUV scale, these images illustrate the characteristic patterns of age-related changes in bone metabolic activity that can be quantitatively assessed in longitudinal studies to monitor physiologic skeletal aging, disease progression, and therapeutic response. Of note, focal areas of increased uptake in the older subject (panel B), particularly in the lumbar spine region, may represent degenerative changes such as facet joint arthropathy rather than generalized age-related metabolic decline. The absence of a standardized grey scale bar in the original publication limits direct quantitative comparison of MIP rendering intensity between the two panels. Readers should therefore interpret the visual differences with caution, and future imaging studies should include standardized SUV color scales to enable accurate inter-subject comparison. Reproduced from Park P.S.U et al. ^18^F-Sodium Fluoride PET as a Diagnostic Modality for Metabolic, Autoimmune, and Osteogenic Bone Disorders: Cellular Mechanisms and Clinical Applications. Int. J. Mol. Sci. 2021 Jun 17;22 [12]:6504., © 2019, with permission under Creative Commons Attribution 4.0International Public License. (https://creativecommons.org/licenses/by/4.0/legalcode) [57]

Interestingly, Park et al. expanded the scope of ¹⁸F-NaF PET/CT utility by exploring its role in monitoring age-related bone turnover changes in a cohort of 88 healthy patients [79]. Analysis of the L1-L4 vertebral trabecular bodies revealed a significant negative correlation between ¹⁸F-NaF SUVₘₑₐₙ and age in females (*P* < 0.0001), with a weaker, but significant, observation in males (*P* = 0.03). In females, ¹⁸F-NaF uptake consistently correlated with age across all acquisition time points. Additionally, in both sexes, ¹⁸F-NaF uptake increased by 10–15% both from 45 to 90 min and from 90 to 180 min post radiotracer injection. These findings emphasize ¹⁸F-NaF PET/CT’s utility for tracking sex-specific changes in vertebral bone turnover and highlight its potential application as a preventive clinical screening tool. These observations are further illustrated in Fig. 1, which displays maximum intensity projection (MIP) ^18^F-NaF PET images comparing skeletal uptake in a young versus older healthy female subject, highlighting regional differences in bone turnover.

It is important to acknowledge that the primary clinical challenge in osteoporosis management is the prediction and prevention of fragility fractures, which represent the most clinically meaningful endpoint. While the aforementioned studies demonstrate that ¹⁸F-NaF PET/CT can detect reduced osteoblastic activity and correlate with DXA-derived BMD, no prospective study has yet directly evaluated the ability of ¹⁸F-NaF PET/CT-derived parameters to predict incident fragility fractures. In contrast, DXA-derived BMD and clinical tools such as FRAX have been extensively validated for fracture risk prediction across large, diverse populations over decades of clinical use [5, 80, 81]. The absence of fracture prediction data for ¹⁸F-NaF PET/CT constitutes a significant gap in the current evidence base and underscores that DXA remains the established reference standard for clinically patent osteoporosis diagnosis and fracture risk stratification. Future prospective studies are critically needed to determine whether ¹⁸F-NaF PET/CT-derived metabolic parameters, either alone or in combination with DXA, can improve fracture risk prediction beyond what is currently achievable with existing clinical tools.

### Role of ^18^F-NaF PET in treatment planning

Beyond its diagnostic utility in lumbar osteoporosis, ¹⁸F-NaF PET has also been explored as a tool for guiding treatment decisions and evaluating therapeutic effects (Table 1). In a cohort of 20 postmenopausal women previously treated with the bisphosphonates alendronate (70 mg weekly, *n* = 11) or risedronate (35 mg weekly, *n* = 9) for a minimum of three years, researchers investigated the impact of discontinuing bisphosphonates on bone metabolism using ¹⁸F-NaF PET [82]. Following cessation of bisphosphonate therapy, a significant reduction in spinal BMD was observed in the alendronate group. Both treatment groups demonstrated significant increases in bone turnover markers by 12 months post-discontinuation. While K_i_ and SUV measurements at the spine and femoral neck did not significantly change, the alendronate group exhibited a notable rise in the SUV at the femoral shaft (33.8%, *p* = 0.028) and total hip (24.0%, *p* = 0.013). These findings by Frost et al. suggest persistent suppression of spinal bone metabolism after bisphosphonate discontinuation and highlight the potential of ¹⁸F-NaF PET in monitoring site-specific skeletal responses. However, the small sample size warrants further investigation to validate these results.

Another study examined the ¹⁸F-NaF kinetics in lumbar spine before and after risedronate treatment [73]. Eighteen postmenopausal women underwent dynamic ¹⁸F-NaF PET scans at baseline and again at six months following therapy. Using the Hawkins model to calculate the net influx rate constant with K_i_, K₁, k₂, k₃, and the dissociation constant from bone (k₄), the study reported a significant 18.4% reduction in mean vertebral K_i_ from baseline, mirroring the decline in bone-specific alkaline phosphatase, which is a marker of bone formation. While K₁ showed no significant changes, a significant increase in k₂ was observed, suggesting enhanced reversal of fluoride transport from the extravascular compartment back into plasma following 6 months of treatment. Despite no significant changes in k₃ and k₄, the fraction of tracer undergoing specific binding to bone decreased by 18.1%, indicating reduced osteoblastic activity.

Uchida et al. used ¹⁸F-NaF PET to evaluate regional bone remodeling changes and their association with conventional bone metabolism markers during alendronate therapy [62]. The study included 24 postmenopausal women with various underlying conditions (excluding rheumatoid arthritis). These patients received ≥ 10 mg oral glucocorticoids daily and were treated with 5 mg/day alendronate for 12 months. ¹⁸F-NaF PET scans were obtained at baseline, 3 months, and 12 months to assess localized bone turnover. Over the 12-month treatment period, alendronate significantly reduced serum bone-specific alkaline phosphatase, urinary N-telopeptide of type I collagen, and SUV values in the lumbar spine and femoral neck, while progressively increasing lumbar spine BMD. Although a significant correlation between BMD and SUV was observed at baseline, this association was no longer present at 12 months. Collectively, the findings in Uchida et al. underscore the utility of ¹⁸F-NaF PET in guiding clinical treatment decisions and monitoring therapeutic response.

While Uchida et al. focused on assessing the clinical response to bisphosphonate therapy, Al-Beyatti et al. aimed to evaluate the reproducibility and reliability of ¹⁸F-NaF parameters in the lumbar spine. The study analyzed measurement precision at the L1-L4 vertebrae in 20 postmenopausal women undergoing bisphosphonate treatment, via alendronate or risedronate, and scanned at baseline, 6 months, and 12 months following therapy discontinuation [74]. Precision errors for SUVs and K_i_ were reported as coefficient of variation (%CV) with 95% confidence intervals. For the L1-L4 region, %CV values were 9.2% for SUV, 11.7% for K_i_ using the Patlak model, and 14.5% for K_i_ using the Hawkins model. No significant difference in precision was observed between the entire L1-L4 spine region and individual vertebrae. Among the parameters, SUV demonstrated the highest reproducibility, followed by K_i_ derived from the Patlak method, while the Hawkins method exhibited the greatest variability. Notably, analyzing smaller anatomical regions did not increase measurement error, suggesting that scanner calibration, rather than region size, was the primary determinant of variability. These findings offer methodological validation that may support the design of future longitudinal studies that aim to monitor treatment response and assess dynamic PET parameters.

Overall, the primary clinical value of ¹⁸F-NaF PET lies in its ability to detect treatment response substantially earlier than conventional imaging modalities. Studies demonstrate significant changes in PET parameters within 3–6 months of therapy, well before BMD changes become apparent on DXA [83, 84]. Frost et al. demonstrated that ¹⁸F-NaF PET detected teriparatide response at 12 weeks across multiple skeletal sites, with Ki increases ranging from 18 to 51% depending on anatomical location [83]. This ability to detect early treatment response enables clinicians to identify non-responders and optimize treatment strategies before investing significant time and resources in ineffective therapies. Consequently, treatment response monitoring rather than primary diagnosis represents the technology’s most valuable clinical application, positioning ¹⁸F-NaF PET as a powerful tool for personalized osteoporosis management in research settings and select clinical scenarios.

Collectively, these studies highlight the clinical use and methodological advantages of ¹⁸F-NaF PET, serving as a crucial tool in guiding physicians for optimizing therapeutic strategies and assessing treatments. Although there are several reviews assessing the role of ¹⁸F-NaF PET in bone disorders, none have comprehensively examined its application in the lumbar spine. Furthermore, our review synthesizes quantitative SUV parameters and kinetic models, allowing for a role in static and dynamic parameters to improve lumbar spine osteoporosis diagnosis and treatment.

### Practical clinical implementation recommendations

Translation of ¹⁸F-NaF PET/CT from research to clinical practice requires clear indications for when this modality adds value beyond standard DXA assessment. Based on current EANM and SNM guidelines, ¹⁸F-NaF PET/CT should be considered complementary to DXA in specific clinical scenarios: (1) early treatment response monitoring, which represents the most clinically impactful application given the modality’s ability to detect metabolic changes within 3–6 months compared to 18–24 months required for DXA to demonstrate significant BMD changes; [62, 73, 82–87] discordant clinical risk and DXA findings, particularly when FRAX scores suggest high fracture risk despite DXA demonstrating only osteopenia, or when patients present with unexplained fragility fractures despite normal or mildly reduced BMD; [80, 81] (2) pre-surgical risk assessment in patients being considered for spinal fusion or vertebral augmentation with borderline bone density measurements; [29] and (3) suspected secondary osteoporosis with complex metabolic derangements where localized assessment may guide targeted intervention, including glucocorticoid-induced osteoporosis, chronic kidney disease-mineral bone disorder, or multiple myeloma [62, 78]. While primary diagnosis of osteoporosis remains dominated by DXA due to cost, radiation exposure, and established clinical thresholds, ¹⁸F-NaF PET/CT’s strength lies in its regional-specific, real-time metabolic assessment that enables personalized treatment decisions and early response evaluation.

Standardized reporting is essential for reproducibility and clinical utility. Reports should specify the acquisition protocol used (static versus dynamic, timing post-injection, reconstruction parameters) and clearly define regions of interest covering L1-L4 vertebral trabecular compartments while excluding posterior elements, cortical shell, and degenerative changes [7, 61, 75, 77–79, 88, 89]. Each vertebral level should be reported individually with aggregate L1-L4 metrics. Quantitative parameters must include SUV_mean_ and SUV_max_ with explicit documentation of normalization method (body weight, lean body mass, or body surface area) and acquisition timing relative to injection [58, 61]. Additionally, diffuse bone hyperactivity may reflect flare phenomenon after chemotherapeutic drug use and other clinical conditions, such as metabolic bone disorders, renal osteodystrophy, and hyperparathyroidism. When kinetic analysis is performed, Ki should be reported with specification of the modeling method used (Patlak or Hawkins), recognizing that Patlak-derived Ki demonstrates superior precision for clinical applications [66, 67, 73, 74]. Hounsfield units from corresponding CT regions should be documented to provide structural context, as HU values show strong correlation with both metabolic parameters and bone mineral density [9, 77, 90, 91].

Critically, reports must include cautionary notes regarding factors that confound interpretation. Active inflammation (facet joint arthritis, spondylitis, spondylodiscitis) produces elevated uptake independent of systemic bone metabolism [92, 93]. Healing fractures demonstrate markedly elevated focal uptake that may persist for months after clinical healing [94]. Malignant involvement including metastases, multiple myeloma, or lymphoma shows increased uptake that may be misinterpreted as increased bone formation [78, 95]. Additionally, degenerative changes including osteophytes and facet sclerosis create focal areas of elevated uptake unrelated to generalized osteoporotic disease [61, 77, 96]. Paget’s disease, recent vertebral augmentation procedures (kyphoplasty, vertebroplasty), and sites of prior radiation therapy all demonstrate altered tracer kinetics [97, 98]. Patient factors, particularly renal function, significantly affect tracer pharmacokinetics and must be documented, as impaired clearance leads to elevated blood pool activity and altered bone-to-background ratios [31, 32]. Reports should explicitly state that ¹⁸F-NaF uptake reflects osteoblastic activity rather than bone mineral density, with the critical caveat that effective anti-resorptive therapy appropriately suppresses bone turnover, resulting in decreased SUV that indicates therapeutic response rather than disease progression, a key interpretive principle distinguishing treatment effect from pathologic bone loss [62, 71, 73, 82, 87]. Lastly, interpreting PET, CT, and DXA findings within clinical context (treatment status, fracture history, comorbidities) with percentage change calculations when comparing serial studies enhances clinical utility and guides management decisions [99].

### Limitations of ¹⁸F-NaF PET

While ¹⁸F-NaF provides valuable information regarding osteoblastic activity and bone turnover, it does not directly measure bone mineral density or yield T-scores. As such, DXA remains the gold standard for osteoporosis diagnosis through its direct quantification of BMD. Additionally, radiotracer uptake is observed in several processes that increase osteoblastic activity, so an increase in uptake in concurrent clinical conditions, such as inflammation, metastasis to the bone, or physiologic healing of fractures, may confound interpretation of ¹⁸F-NaF imaging. Additionally, there are challenges associated with standardization between institutional protocols, and any differences in parameters like acquisition timing or image protocols can affect quantitative parameters such as SUV. Furthermore, renal impairment and limited renal clearance, seen in patients with kidney disease, can alter radiotracer pharmacokinetics and systemic availability, so patient comorbidity can limit interpretation of ¹⁸F-NaF PET results. PET/CT has limitations to access due to its higher cost, and so there is limited availability across institutions. Finally, ¹⁸F-NaF carries higher risks of radiation, so it may not be preferred for serial monitoring over time, unlike a modality with lower radiation risks such as DXA.

Given these respective strengths and limitations, the clinical preference between DXA and ¹⁸F-NaF PET/CT should be guided by the specific clinical question. DXA should remain the preferred modality for initial osteoporosis screening, routine population-based assessment, longitudinal BMD monitoring in standard clinical settings, and situations where cost and radiation minimization are paramount. ¹⁸F-NaF PET/CT should be preferred when early detection of treatment response is needed (within 3–6 months rather than the 18–24 months required for DXA), when there is clinical suspicion of regional metabolic abnormalities not captured by areal BMD measurements, and in pre-surgical planning where site-specific bone quality assessment is critical. Both modalities should be utilized together when clinical risk assessment and DXA-derived BMD are discordant (e.g., fragility fractures occurring despite normal or mildly reduced T-scores), in complex metabolic bone disorders such as glucocorticoid-induced osteoporosis or renal osteodystrophy where both structural and metabolic information is needed, and in research settings where comprehensive characterization of bone health is required. Ultimately, ¹⁸F-NaF PET/CT should be viewed as a complementary tool that addresses the diagnostic gaps of DXA rather than as a replacement.

## Conclusion

Based on available evidence, ¹⁸F-NaF PET/CT should be considered complementary to DXA rather than a replacement. Its optimal clinical applications include early treatment response monitoring, evaluation of patients with discordant clinical risk and DXA findings, pre-surgical assessment in patients with borderline bone density, and investigation of complex metabolic bone disorders. As healthcare increasingly emphasizes personalized medicine, the ability of ¹⁸F-NaF PET/CT to provide individualized, site-specific assessment of bone metabolism positions it as a valuable tool for optimizing osteoporosis management. However, larger prospective validation studies, cost-effectiveness analyses, and standardized interpretation criteria remain necessary before routine clinical implementation can be recommended for general populations.

## Data Availability

Not Applicable.

## Abbreviations

AUROC: Area under the receiver operating characteristic
BMAD: Bone mineral apparent density
BMD: Bone mineral density
CT: Computed tomography
DXA: Dual-energy X-ray absorptiometry
FDA: U.S. Food and Drug Administration
HU: Hounsfield units
K₁: Plasma to bone transfer rate constant
k₂: Bone to plasma efflux rate constant
k₃: Bone binding rate constant
k₄: Dissociation rate constant from bone
Ki: Net influx rate constant
MIP: Maximum intensity projection
mSv: Millisievert
PET: Positron emission tomography
QCT: Quantitative computed tomography
SPECT/CT: Single-photon emission computed tomography/computed tomography
SUV: Standardized uptake value
SUVₘₐₓ: Maximum standardized uptake value
SUVₘₑₐₙ: Mean standardized uptake value
⁹⁹ᵐTc-MDP: Technetium-99 m methylene diphosphonate
¹⁸F-NaF: Fluorine-18 sodium fluoride
